# Prion protein cleavage fragments regulate adult neural stem cell quiescence through redox modulation of mitochondrial fission and SOD2 expression

**DOI:** 10.1007/s00018-018-2790-3

**Published:** 2018-03-24

**Authors:** Steven J. Collins, Carolin Tumpach, Bradley R. Groveman, Simon C. Drew, Cathryn L. Haigh

**Affiliations:** 1 Department of Medicine (Royal Melbourne Hospital), The University of Melbourne, Melbourne, VIC 3010 Australia; 2 Doherty Institute, The University of Melbourne, Melbourne, VIC 3010 Australia; 3 Laboratory of Persistent Viral Diseases, Rocky Mountain Laboratories, NIAID, NIH, Hamilton, 59840 USA

**Keywords:** NADPH oxidase, Nox2, DRP1, Superoxide dismutase, SOD2, Reactive oxygen species, Mitochondria, N1, N2

## Abstract

Neurogenesis continues in the post-developmental brain throughout life. The ability to stimulate the production of new neurones requires both quiescent and actively proliferating pools of neural stem cells (NSCs). Actively proliferating NSCs ensure that neurogenic demand can be met, whilst the quiescent pool makes certain NSC reserves do not become depleted. The processes preserving the NSC quiescent pool are only just beginning to be defined. Herein, we identify a switch between NSC proliferation and quiescence through changing intracellular redox signalling. We show that N-terminal post-translational cleavage products of the prion protein (PrP) induce a quiescent state, halting NSC cellular growth, migration, and neurite outgrowth. Quiescence is initiated by the PrP cleavage products through reducing intracellular levels of reactive oxygen species. First, inhibition of redox signalling results in increased mitochondrial fission, which rapidly signals quiescence. Thereafter, quiescence is maintained through downstream increases in the expression and activity of superoxide dismutase-2 that reduces mitochondrial superoxide. We further observe that PrP is predominantly cleaved in quiescent NSCs indicating a homeostatic role for this cascade. Our findings provide new insight into the regulation of NSC quiescence, which potentially could influence brain health throughout adult life.

**Electronic supplementary material:**

The online version of this article (10.1007/s00018-018-2790-3) contains supplementary material, which is available to authorized users.

## Introduction

It is now firmly established that the adult brain contains cells that demonstrate ‘stemness’, i.e., are capable of self-renewal and formation of new brain cells (reviewed in [1]). Neural stem cells (NSCs) have been detected in human brain tissue from donors up to the age of 72 years [2] and markers of neurogenesis detected into the ninth decade [3], indicating the likely importance of these cells throughout adult life. The adult brain contains different populations of NSCs. Type 1 NSCs are quiescent and can be stimulated to become type 2 NSCs, which are actively replicating. Type 2 cells in turn progress to be type 3 migratory cells or neuroblasts [1, 4]. NSC growth and differentiation have been linked with hippocampal learning and memory as well as forgetting [5–9], and changes in neurogenesis correlate with various mental health issues and neurodegenerative diseases [3, 10].

Neurogenesis is modulated by several neurodegenerative disease-associated proteins or peptides including the prion protein (PrP) [11, 12], which is most widely recognised for its causative role in transmissible neurodegenerative diseases of humans and animals [13]. Research into the role of PrP in neurogenesis has found that PrP expression is linked with enhanced NSC proliferative capacity [12], associated with increased cell cycling at the expense of differentiation [14]. In addition, PrP has been shown to be part of a receptor complex for soluble neurotoxic Alzheimer’s disease-associated amyloid-beta peptides [15, 16] and the presence or absence of PrP changes NSC self-renewal in response to amyloid-beta peptides [17], thereby suggesting that a putative neurogenic function of PrP may become corrupted during neurodegenerative disease.

**Fig. 1:**
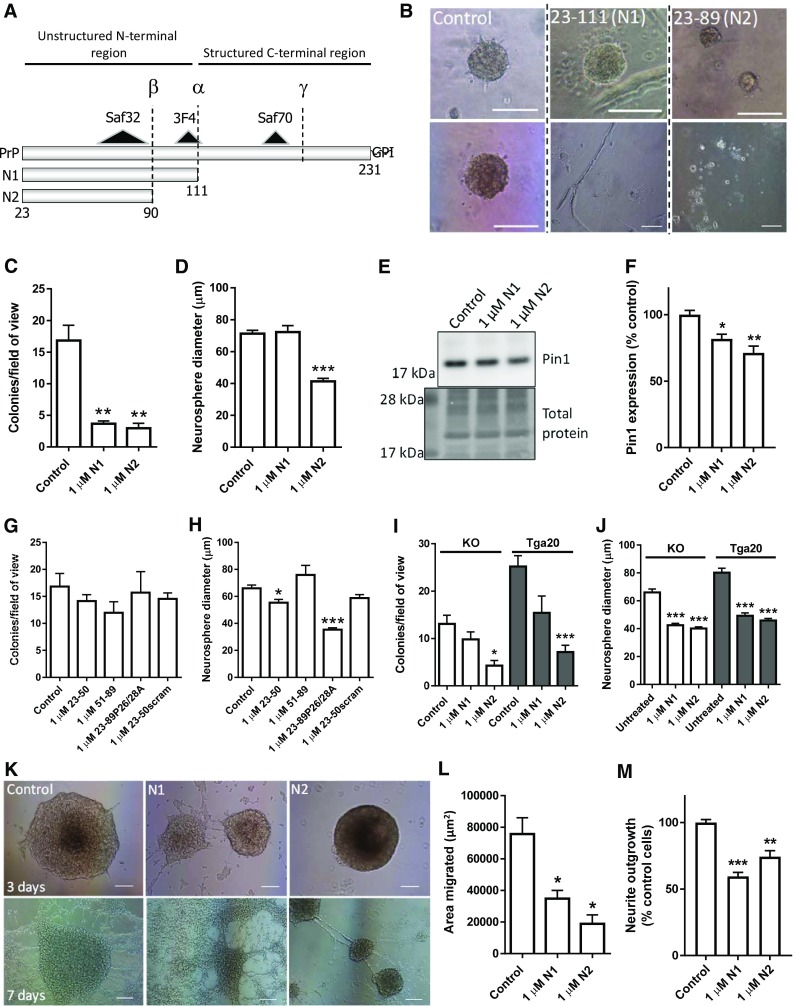
N1 and N2 change NSC growth, migration, and neurite outgrowth. **a** Schematic of PrP cleavage events, the cut positions producing the N1 and N2 fragments, and the putative site of gamma-cleavage. Also shown are the epitopes of the antibodies used to target PrP. **b** Representative images of neurosphere colony morphology in the NCFA with and without peptide treatments. Two images each are shown to illustrate the varied morphology. Scale bar = 100 μm. **c** NCFA colony counts of NSCs incubated with and without N1 and N2 included in the assay matrix. *n* = 4. **d** Diameter measurements of colonies formed in the NCFA with and without peptide incubation. *n* = 4. **e** Immunoblots for Pin1 of NSCs treated for 24 h with N1 or N2. **f** Densitometric quantification of **e**. *n* = 4. **g** NCFA colony counts of NSCs incubated with fragments corresponding to regions of N2 and an N2 peptide with the prolines of the N-terminal polybasic region mutated to alanine. *n* = 3. **h** Diameter measurements of the NCFA described in G. *n* = 3. **i** NCFA colony counts of N1 and N2 incubated with *prnp* knock-out (KO) and over-expressing (Tga20) NSCs. *n* = 3. **j** Diameter measurements of the NCFA described in I. *n* = 3. **k** Representative images of neurosphere migration as influenced by N1 and N2 taken 3 and 7 days after peptide addition. Scale bar = 100 μm. **l** Quantification of the area migrated after 3 days incubation as shown in **k**. *n* = 3. **m** Neurite outgrowth of NSCs incubated with N1 and N2 relative to control cells. *n* = 3. All data within the figure panels are presented as mean and SEM with significance indicated by *p < 0.05, **p < 0.01, ***p < 0.001 from control values

Despite much interest in the role of PrP during disease, there is still no defined function for this protein. However, PrP is known to be involved in cellular redox balance, both protecting against detrimental disturbance in health and causing damage during prion disease [18–27]. The cellular biology of PrP is complex; mature PrP is a glycosylphosphatidylinositol membrane-tethered glycoprotein, known to undergo various other post-translational modifications including internal cleavage at three or more sites, termed alpha-, beta- and gamma-cleavages [28–30]. The alpha- and beta-cleavage events remove the flexible, unstructured N-terminus from the structured C-terminal region, producing N1/C1 and N2/C2 fragments, respectively (Fig. 1a). Various cellular locations of cleavage have been proposed including the secretory pathway and cell surface, and the N-terminal fragments have predominantly been found as extracellular soluble peptides [31, 32]. As alluded to above, PrP is associated with cellular redox homeostasis and the N1 and N2 fragments alone have been shown to reduce intracellular reactive oxygen species (ROS) and protect against oxidative damage [32, 33].

Redox balance has a regulatory role in NSC growth and differentiation [34, 35]. Adult NSCs are not dispersed evenly through the brain but situated in specialised regions, referred to as neurogenic niches [36]. In the neurogenic niche, the environment is highly important for the preservation of the NSC populations and is maintained as a low ROS environment [37]. Increased NSC ROS are associated with stimulation of increased growth, possibly at the expense of depleting the quiescent pool in favour of the actively proliferating cells [34, 35, 38]. In neurodegenerative diseases, such as prion diseases and Alzheimer’s disease, where redox balance is compromised resulting in increased ROS, increased markers of neurogenesis are detected in brain tissue [39]. We hypothesized that the ability of the N-terminal PrP peptides, N1 and N2, to modulate cellular ROS levels could exert significant regulatory effects on NSC growth and maintenance of the quiescent NSC pool.

## Methods

### Prion N-terminal peptides

The prion N-terminal peptides were generated by microwave synthesis and quality controlled by HPLC and mass spectrometry as previously described in Karas et al. [40]. Peptides were also purchased from China Peptides (China).

### Adult neural stem cell extraction

NSCs were harvested from the brains of three 8-week-old PrP gene (*prnp*)^−/−^ (PrP knock-out), C57/B6 wild-type, and Tga20 (PrP over-expressing by approximately eightfold) mice, and transferred into suspension culture as described previously in Haigh et al. [24]. All animal procedures were performed in accordance with University of Melbourne animal ethics committee approval (ethics ID: 1413198.1), operating under the Australian Code of Practice for the Care and Use of Animals for Scientific Purposes.

### NSC culture

Routine NSC culture has been described previously [24]. In brief, cells were grown as neurospheres in complete proliferation medium (Stem Cell Technologies, VIC, AUS), supplemented with final concentrations of 10 ng/ml fibroblast growth factor (FGF) and 20 ng/ml epidermal growth factor (EGF) cytokines, and 2 μg/ml heparin (Stem Cell Technologies). Cultures were maintained in a 5% CO_2_ humidified incubator.

### Cell counts

Cells were mixed 1:1 (v/v) with Trypan Blue reagent (Life Technologies, VIC, AUS) and counted using a Countess cell counter (Life Technologies).

### Neural colony forming assay (NCFA)

The colony forming assay is described in Collins et al. [17]. Briefly, neurospheres are dissociated into single-cell suspension and seeded in a semi-solid gel matrix made with a 2:1 solution of proliferation medium and collagen (Sigma-Aldrich, VIC, AUS) in 24-well plates at a density of 50,000 cells/ml (25,000 cells/well). Wells were supplemented with 80 μl of fresh proliferation medium every 5 days. Treatments were included at the start of the assay only. Colonies were counted and diameters measured at 21 days post the start of the assay. All colonies at all depths through the matrix were calculated and measured for a minimum of 5 fields or 50 colonies (for conditions where few colonies were found per field of view).

### Migration assay

Neurospheres of 80–100 μm diameter were seeded into wells pre-coated with 5 μg/ml poly-d lysine (PDL; Sigma-Aldrich) in H_2_O solution (for a minimum of 1 h at room temperature) and grown in complete proliferation medium. After 3 days, migration from the central neurosphere was calculated by application of contour ROIs around the surface area into which the cells had spread using NIS Elements 3.0 (Nikon) software package. The area covered after 7 days could not be calculated as migration had proceeded beyond the imaging area.

### Neurite outgrowth assay

One hundred thousand cells/well were cultured in complete proliferation medium supplemented with 20 ng/ml FGF for 24 h before transferral into complete differentiation medium (Stem Cell Technologies). The neurite outgrowth assay (Millipore, Thermo-Fisher, VIC, AUS) was carried out as per the manufacturer’s instructions. Well inserts (1 µm pore size) were coated on their under-side with 10 µg/ml laminin in H_2_O solution for 2 h at 37 °C before NSCs were transferred into the insert. Cells were cultured for 2 days in differentiation medium before assaying outgrowth. To assay neurite extension onto the laminin coated under-side, well inserts were first washed in PBS, then fixed in methanol for 20 min at room temperature, washed again by transferring the insert into PBS, and then stained with neurite stain solution for 30 min. Inserts were washed again in PBS, excess dye removed from the inside of the insert, and cell somas removed by swabbing the inside of the insert. The neurites extending over the under-side of the well insert were then solubilised in extraction buffer and the absorbance read in a Spectrostar (BMG) at 562 nm. Neurite outgrowth, indicated by absorbance of the neurite stain as compared with extraction buffer alone, was calculated using MARS software (BMG).

### Immunofluorescence staining

The standard immunofluorescence protocol used has been described previously [24, 41]. Briefly, cells were fixed in 4% (v/v) paraformaldehyde for 30 min, followed by permeabilization in 0.1% triton-X-100, before blocking in 10% (v/v) FBS, 0.1% (w/v) BSA in PBS for 30 min. Primary and secondary antibody incubations were carried out in 1% (v/v) FBS, 0.1% (w/v) BSA in PBS blocking buffer, with details and relevant concentrations listed in Sup Table 1.

### Microscopy

Confocal images were collected using a Leica SP8 (Leica Microsystems, NSW, AUS) and wide-field fluorescence or bright-field images captured using a Nikon Eclipse TE2000-E epi-fluorescence microscope with a Roper Scientific CCD camera (Nikon, Coherent Scientific, SA, AUS). Image analysis was performed using Fiji imaging software [42] or NIS Elements 3.0 (Nikon). Mitochondrial parameters (minimum of six fields from three independent experiments) were calculated using the mito-morphology macro for image J created by Ruben K. Dagda at the University of Pittsburgh. Image enhancements, despeckling, were performed for inset digital zooms to increase picture clarity.

### Pharmacological inhibition of pathway activity

Diphenyleneiodonium chloride (DPI; Sigma-Aldrich) stocks were prepared in water and stored at room temperature. N-[2-(*p*-Bromocinnamylamino)ethyl]-5-isoquinolinesulfonamide dihydrochloride (H89; Abcam, VIC, AUS) stocks were prepared in water and stored at -20°C.

### Cell plating

Cells were seeded at a density of 3.6 × 10^4^ cells/well in 5 μg/ml PDL in H_2_O solution-coated 96-well plates and allowed to adhere under normal incubator conditions for 24 h before assay.

### [3-(4,5-Dimethylthiazol-2-yl)-5-(3-carboxymethoxyphenyl)-2-(4-sulfophenyl)-2H-tetrazolium (MTS) metabolism assay

Five microlitres of one-solution MTS reagent (Promega, VIC, AUS) was added per 100 μl of media in each well. Plates were incubated under normal incubator conditions for the duration of the assay. Developed colour was measured at 492 nm in a FluoSTAR/PolarSTAR Optima (BMG Labtech, VIC, AUS).

### Lactate dehydrogenase (LDH) cell death assay

Cellular and extracellular LDH was measured using a Cytotoxicity Detection kit (LDH; Roche, VIC, AUS) and cytotoxicity calculated as per the manufacturer’s instructions.

### Caspase 3/7 assay

Caspase 3/7 staining and 7-amino-actinomycin D (7-AAD) staining were carried out and quantified using a Muse™ Caspase-3/7 Kit (Roche-Sigma, USA) and a Muse™ Cell Analyzer (Roche-Sigma) with software version 1.4. The cells were loaded as per the manufacturer’s instruction with 5 µl of Caspase-3/7 working solution added to 50 µl cells (diluted to approximately 1 × 10^6^ cells/ml). Cells were incubated for 30 min at 37 °C before addition of 150 µl of 7-ADD working solution and then analysed for apoptotic (but not dead) and dead cells.

### Ki67 assay

Ki67 was assayed using the Muse^®^ Ki67 Proliferation Kit (Roche-Sigma) and a Muse™ Cell Analyzer (Roche-Sigma). Cells were fixed and stained as per the manufacturer’s instructions and the negative control staining (shown in grey shading on the plots) was used to gate the background unstained cell population for analysis using the Muse v1.4 software.

### Adenosine triphosphate (ATP) assay

Cellular ATP content was measured by luminescence using Life Sciences ATP assay (Life Technologies) as per the product protocol and normalised for total protein levels determined by BCA assay (Pierce, Thermo-Fisher).

### Dichlorofluorescin diacetate (DCFDA) assay

The DCFDA assay for intracellular ROS production has been described previously [33]. Briefly, cells were loaded with CM-H_2_-DCFDA reagent (Invitrogen) by incubating cells with a 5 µM probe solution in PBS for 20 min at 37 °C. Basal fluorescence was then read to provide a well-background control and test conditions added to start the assay. Fluorescence intensity was monitored for 12 h.

### Nicotinamide adenine dinucleotide phosphate (NADPH) consumption assay

NADPH (Arbor Assays, Bio Scientific, VIC, AUS) was diluted to 250 mM stock in sterile water, and aliquoted and stored at – 20 °C until use. At the start of the assay, 1 μM peptide was included in the culture media; then, 25 mM NADPH was added to each well and decay of the NADPH absorbance monitored at 340 nm every 60 s for 10 min and gradients calculated to show consumption of substrate.

### Superoxide dismutase (SOD) activity assay

Total SOD activity of cell lysates was determined using a WST-1-based competitive inhibition assay (Abcam), as per the manufacturer’s instructions, with normalisation of activity to total protein as determined by BCA assay.

### Peptide:*N*-glycosidase F (PNGase-F) digests

Pre-denatured lysates were digested using 1 µl PNGase-F per 20 µl protein sample with overnight incubation at 37 °C.

### Western/dot blotting

Western blotting was carried out using the Invitrogen NuPAGE/Bolt gel system (Life Technologies) with Criterion (BioRad, VIC, AUS) or iBlot (Invitrogen) transfer and developed as described previously [41, 43]. Coomassie membrane staining was performed as previously described [44]. Total protein staining was used to monitor gel loading/transfer efficiency, because the housekeeping proteins often used as loading controls are involved in the cellular processes being investigated and are, therefore, considered unreliable. Dot blotting was performed by dotting 2 μl of whole-cell lysate onto nitrocellulose membranes. Membranes were blocked and blotted as for standard western blotting procedure. Antibody information is shown in Sup Table 1. Percentage change was calculated by first normalising band signal to total protein and then applying the following equation:  % change = (test band signal intensity/control band signal intensity) × 100.

### Senescence staining and quantification

Senescence was determined using a β-galactosidase staining kit (Cell Signaling Technologies, Sapphire Biosciences, VIC, AUS), following the manufacturer’s instructions and the blue product solubilised in DMSO by agitated heating as described in Haigh et al. [44].

### Redox-sensor Red ROS assay

Redox-sensor Red (PF-H_2_TMRos; Life Technologies) at a final concentration of 5 μM in Opti-MEM I reduced serum culture medium (phenol red-free; Invitrogen) was loaded into cells for 10 min and cells were imaged in fresh medium as described previously [24].

### MitoSOX staining

MitoSOX fluorescent indicator probe (Life Technologies) was loaded into cells at a final concentration of 5 μM in normal media for 10 min under standard incubator conditions and imaged in fresh, phenol red-free Opti-MEM as described previously [45].

### Small interfering RNA (siRNA) transfections

Pre-validated siRNA duplexes were purchased from Life Technologies. Single-cell suspensions were prepared in proliferation media and sufficient cells seeded into 1-well of a 6-well plate for all test and control assays. Transfections were achieved using Fugene HD transfection reagent (Roche) as per the manufacturer’s protocol. Plates were returned to the incubator until cells were used for assay. Prior to assay, cells were re-suspended as single cells, counted and seeded into the NCFA as described previously. Pre-screening of knock-down efficiency found that knock-downs of both Nox2 and SOD2 were optimal (~ 50%) at 2 days using 30 μM siRNA for Nox2 and 50 μM for SOD2 (example western blot images of knock-downs are shown in Sup Fig 1). Therefore, all assays began 2 days post-treatment with these concentrations of siRNA and the equivalent of non-silencing control (Life Technologies). At the start of every assay, a dot blot was performed on cells from the starting culture to confirm the knock-down had been consistent. Quantifications of these quality control spots are shown in Sup Fig 1.

### Statistical analysis

Statistical analyses were accomplished using GraphPad Prism 5 statistical software. Students *t* tests were used for comparison of two parameters and ANOVA or Kruskal–Wallis analyses used for > two parameters. Where significant differences were found, Dunnett, Bonferroni, or Dunn tests were used for multiple comparisons of one-way, two-way, and non-parametric ANOVA, respectively. *p *< 0.05 was used as the cutoff for significance and ≥ 95% statistical power. All stated “*n*” values indicate independent repeats.

## Results

### N1 and N2 alter NSC growth

To assess the ability of the soluble N-terminal fragments to influence NSC growth, adult NSCs (harvested from wild-type mice at 8 weeks of age) were incubated with 1 μM synthetically produced N1 and N2 peptides (Fig. 1a) included in the matrix of a neural colony forming assay (NCFA; Fig. 1b). The 1 μM concentration of each peptide was based upon our previous studies showing functionality at these concentrations [33, 41, 46]. Exposure of the NSCs to either the N1 or the N2 fragment resulted in decreased growth, reducing the number of colonies formed (Fig. 1c) and, for the N2 fragment, the diameter of the colonies (Fig. 1d). The N2 growth reduction was accompanied by a significant reduction in protein expression of the cell proliferation regulatory protein Pin1 (Fig. 1e, f). Using further synthetic fragments corresponding to shorter regions of the N2 fragment, and also an N2 peptide with mutated N-terminal residues, it was found that wild-type full-length N2 is the minimum needed to cause overall growth change, especially with respect to colony formation (Fig. 1g, h). A fragment representing the most N-terminal region, amino acids 23–50, could elicit a small effect on colony diameter, but, whilst this was significantly different from the untreated control, it was not statistically different from a scrambled control peptide suggesting an artefactual result (Fig. 1h). The consequence of N-terminal mutation was less clear. Whilst the effect of wild-type N2 on reducing the number of colonies formed was clearly abolished by mutation of the N-terminal proline residues to alanine (P26/28A), there was a persisting capacity to reduce colony diameter similar to that observed with un-mutated N2 fragment.

During prion disease, ongoing PrP expression is an absolute requirement for pathogenesis [47]. Therefore, we investigated whether the influence of the N1 and N2 peptides on cell growth was changed in cells of differing PrP expression levels. The effect of the fragments, especially for N2, did not depend upon the underlying expression of PrP in the NSCs, with both the *prnp* knock-out (KO) and Tga20 over-expressing cells showing reduced growth when the peptides were included in their matrix (Fig. 1i, j). However, in contrast with the wild-type cells, the KO and Tga20 cells demonstrated a changed influence of the N1 peptide, with colony diameter more influenced than the number of colonies formed.

### N1 and N2 reduce migration and neurite outgrowth

Other processes that occur following division in actively replicating NSCs include migration of cells to their site of integration and the extension of neurite outgrowths, and both of these processes have been found to be influenced by cellular PrP expression levels [48, 49]. Congruent with the colony forming assay results, both migration and neurite outgrowth were reduced by the N1 and N2 peptides (Fig. 1k–m). By observing the migration of cells from the neurospheres for longer, it was apparent that the inhibitory effects of N1 and N2 were transient with migration of the N1-treated cells indistinguishable from control cells and migration resumed, albeit at an attenuated level, for N2 by 7 days (Fig. 1k).

### N1 and N2 do not cause cytotoxicity or senescence

**Fig. 2:**
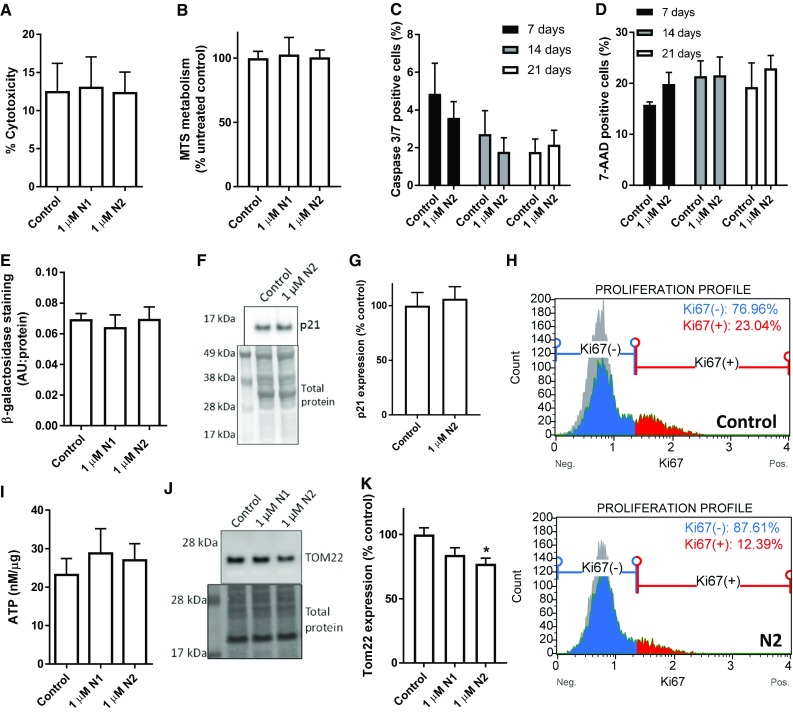
Reduction in growth is not due to reduction in cell viability. **a** Cytotoxicity of N1 and N2 as measured by cellular LDH release 24 h post-exposure. *n* = 4. **b** MTS measurement of cellular metabolism as an indicator of viability 24 h post-exposure to N1 or N2. *n* = 4. **c** Active caspase 3/7 detection in cells cultured for 3-week post-treatment with N2. *n* = 3. **d** Uptake of 7-AAD as an indicator of dead cells in the same time series as c. *n* = 3. **e** Beta-galactosidase staining intensity, as an indicator of cell senescence, 3-day post-exposure to N1 or N2. *n* = 3. **f** Immunoblots for the cell quiescence/senescence-associated protein p21. **g** Densitometric quantification of **f**. *n* = 3. **h** Ki67 flow cytometry analysis of proliferating cells 3 days following treatment with the N2 peptide. Representative plots from *n* = 4. **i** Cellular ATP concentration relative to total protein 24 h after exposure to N1 or N2. *n* = 4. **j** Immunoblots for the mitochondrial transporter protein TOM22 24 h after exposure to N1 or N2. **k** Densitometric quantification of e. *n* = 4. Data are presented as mean and SEM. Significance is indicated by *p < 0.05

To ascertain whether cell death was the cause of the reduced NSC growth in response to the N1 and N2 peptides, cytotoxicity and cell metabolism assays were performed (Fig. 2a, b) after 24 h, which found no discernible changes. To ensure that death was not delayed or increased over the time of the NCFA and migration assays, caspase 3 and 7 (executioner caspase) activation and cell death as indicated by uptake of 7-AAD were monitored weekly using the more potent N2 fragment. These measurements also found no significant effect on long-term viability as a result of peptide exposure (Fig. 2c, d). In addition, beta-galactosidase staining, an indicator of cellular senescence, was not increased in these cells (Fig. 2e). Assessment of the, quiescence/senescence-associated marker p21 showed no change in response to N2 treatment over 3 days (Fig. 2f, g); however, Ki67, a marker of cell proliferation, was reduced to half of the levels detected in control cells (*p* = 0.041, *n* = 4; Fig. 2h). A change in growth might indicate perturbed cellular energy demands; therefore, cellular ATP and mitochondrial protein expression levels were examined. Despite no changes in cellular ATP levels (Fig. 2i), a small decrease in the mitochondrial transporter TOM22 was detected following 24 h exposure to the N2 fragment (Fig. 2j, k), which indicated that mitochondrial mass was influenced by this peptide.

### N1 and N2 reduce intracellular ROS production

**Fig. 3:**
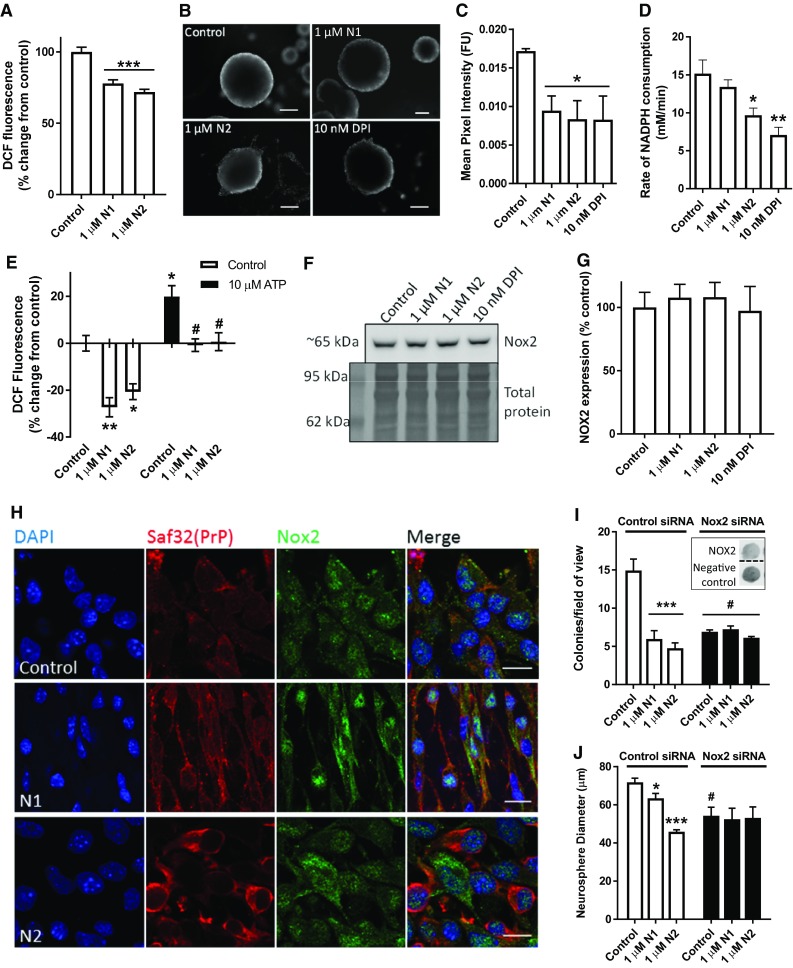
N1 and N2 reduce intracellular redox signalling through the Nox pathway. **a** DCFDA assay detection of intracellular ROS production in NSCs treated with N1 and N2. *n* = 6. **b** Representative images of Redox-Sensor Red staining of whole neurospheres 90 min after exposure to N1, N2 or the Nox inhibitor DPI. Scale bar = 50 μm. **c** Quantification of fluorescence intensity in B. *n* = 3. **d** Cellular NADPH consumption rate within 20 min following exposure to the peptides. *n* = 3. **e** DCFDA assay detection of intracellular ROS production in NSCs treated with N1 and N2 with or without the inclusion of ATP in the assay media. *n* = 4. Hash denotes significantly different from the equivalent peptide condition without ATP. **f** Immunoblots for cellular expression of Nox2 24 h following exposure to the N1 and N2 peptides. **g** Quantification of E. *n* = 4. **h** Representative confocal images of Nox2 cellular staining 90 min after peptide treatment. Scale bar = 10 μm. **i** Colony counts of the NCFA where NSCs were incubated with and without N1 and N2 and with siRNA targeting Nox2 expression or a control non-silencing siRNA (inset shows dot blots of remaining Nox2 expression in a representative experiment). *n* = 3. Hash significantly different from non-silencing siRNA control, *p *< 0.05. **j** Diameter measurements of colonies formed in the NCFA described in **i**. *n* = 3. Hash significantly different from non-silencing siRNA control, *p *< 0.05. All data within the figure are presented as mean and SEM with significance indicated by *p < 0.05, **p < 0.01, ***p < 0.001 from respective control values unless otherwise stated

In our previous studies, we found that the N2 fragment could protect secondary cell lines from oxidative stress and similar protective actions have been reported for the N1 fragment [32, 33, 41]. In addition, redox signalling is known to alter NSC growth [34, 35, 44]. To determine whether the influence of the N1 and N2 peptides on redox balance might underpin the NSC growth changes, we analysed the production of intracellular ROS using the DCFDA assay. The DCFDA assay is a fluorometric assay wherein cells are loaded with DCFDA; the intracellular form of DCFDA, DCF^−^, is non-fluorescent until it is oxidised to DCF by free radicals, and therefore, an increase in fluorescence signal over the control cells indicates increased radical production and vice versa. Performing the DCFDA assay found a significant reduction of ROS production in cells incubated with either peptide (Fig. 3a). We have previously found that inhibiting NADPH oxidase (Nox) activity using the pharmacological inhibitor DPI reduces NSC growth in the NCFA [44]. Therefore, intracellular ROS levels were further considered in the NSCs using Redox-Sensor Red. Redox-Sensor Red is a partitioning probe; fluorescing when it binds ROS in the cytosol, which are primarily produced by the Nox family, or, if cytosolic ROS are not met, it then accumulates in lysosomes where its fluorescence increases as influenced by lysosomal redox balance. Redox-Sensor Red was loaded into whole neurospheres prior to addition of N1, N2, or DPI and spheres were imaged at 90 min. Fluorescence intensity showed a similar significant reduction in ROS detected for the N1-, N2-, and DPI-treated conditions (Fig. 3b, c). We next measured cellular usage of NADPH, finding that N2 and DPI showed a significant decrease in the utilisation of NADPH indicating a lesser activity of this family of enzymes (Fig. 3d). To further assess the potential for N1 and N2 to modulate Nox family function, we competed the peptides against ATP, which activates Nox signalling increasing cellular ROS production, and used the DCFDA assay to monitor changes within the cells. The peptides were overlaid on the cells first and then ATP was included in the assay media. ATP increased cellular ROS production significantly from the basal control and both peptides were able to inhibit this effect (Fig. 3e).

To directly investigate the role of Nox signalling, siRNA knock-down was used to target Nox2. Nox2 was selected as it is a central catalytic subunit of the Nox complex and known to have a role in the maintenance of NSC populations within the brain [50]. Nox2 has also previously been linked with PrP signal transduction and to prion disease pathogenesis [51–53]. Western blotting confirmed that the stem cells do express this subunit and showed no significant changes in the expression levels of Nox2 after 24 h incubation with N1, N2 or DPI (Fig. 3f, g). Confocal imaging of PrP and Nox2 showed altered cellular morphology and localisation of Nox in response to the peptides (Fig. 3h). Using siRNA to knock down the expression of Nox2, a significant decrease in cellular growth was seen in the NCFA (consistent with the previously reported effects of DPI treatment) and this completely negated the effects of both N1 and N2 (Fig. 3i, j).

### Reduced redox signalling causes increased mitochondrial fission

**Fig. 4:**
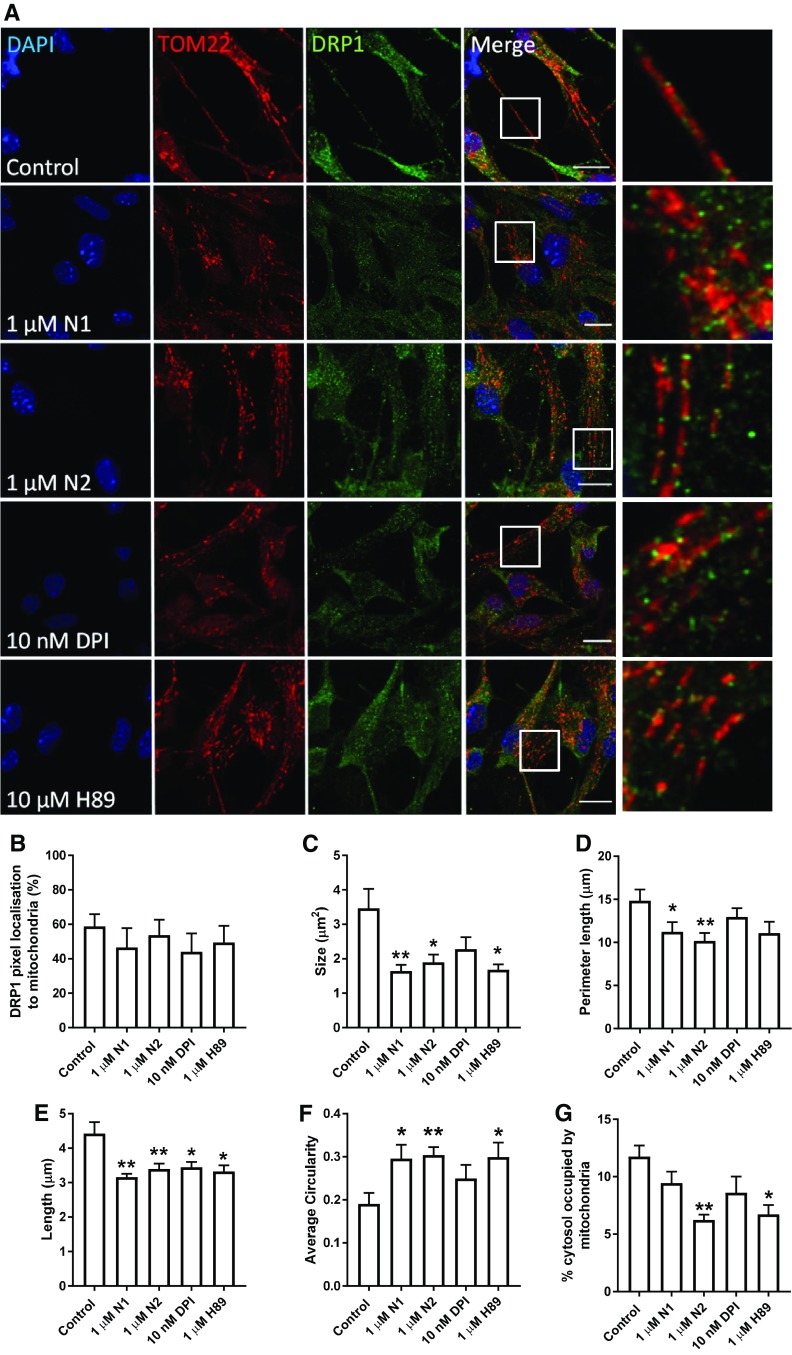
N1 and N2 increase mitochondrial fission. **a** Representative confocal images of DRP1 and mitochondrial (TOM22) morphology 90 min following addition of N1, N2, DPI, and PKA inhibitor H89 to NSCs. Scale bar = 10 μm. Boxes indicate the region shown in higher magnification on the right. **b** Measurement of percentage DRP1 stained pixels localising within mitochondria. *n* = 3. **c** Measurement of mitochondrial size under the conditions described in **a**. *n* = 3. **d** Measurement of mitochondrial perimeter length under the conditions described in **a**. *n* = 3. **e** Measurement of mitochondrial length (measurement of longest axis) under the conditions described in **a**. *n* = 3. **f**. Measurement of mitochondrial circularity under the conditions described in **a**. *n* = 3. **g** Measurement of cytosolic  % occupied by mitochondria under the conditions described in **a**. *n* = 3. All data within the figure are presented as mean and SEM with significance indicated by *p < 0.05, **p < 0.01 from respective control values

**Fig. 5:**
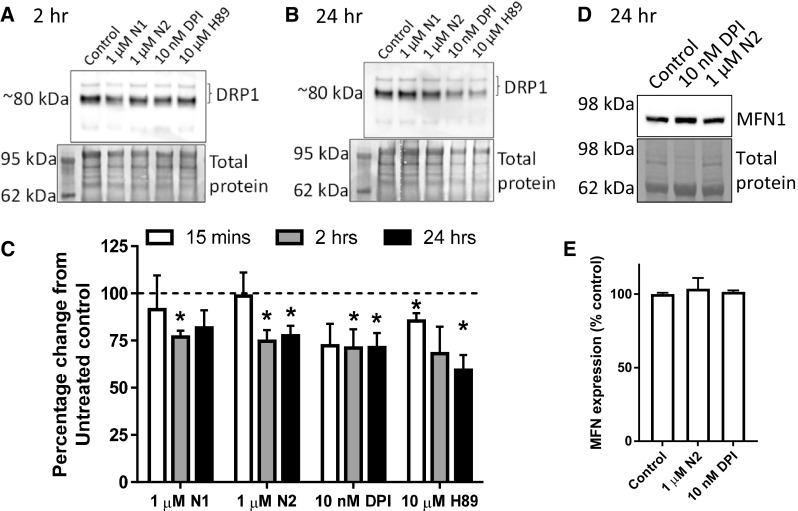
N1 and N2 change DRP1 detection but not MFN1. **a** Immunoblots of DRP1 2 h following addition of N1, N2, DPI, and PKA inhibitor H89 to NSCs. **b** Immunoblots of DRP1 24 h post-treatment as described in **a**. **c** Densitometric quantification of DRP1 immunoblots at 15 min, 2 h  (**a**) and 24 h (**b**). *n* = 4. **d** Immunoblots of MFN1 24 h post-treatment with N2 or DPI. **e** Densitometric quantification of **d**. *n* = 3. All data within the figure are presented as mean and SEM with significance indicated by *p < 0.05 from respective control values

We next wished to determine how N1/N2-redox signalling could be transduced through the cell to change cell growth. Nox signalling is linked with many of the central signal transduction pathways of the cell. We investigated several of these, including ERK1/2, AKT1, GSK-3β, and p38, but found no obvious activation or deactivation of any of those tested (Sup Fig 2). Since the central cellular signalling pathways were not seen to be influenced by the peptides, but mitochondrial involvement was indicated by TOM22 reduction in response to N2 (Fig. 2j, k), direct changes to mitochondria were considered. Mitochondrial fission and fusion are linked with cell cycle control [54]. Furthermore, in times of heighted oxidative stress, including that caused by Nox and exogenous H_2_O_2_, mitochondria fuse to form long networks protecting themselves from autophagy [55]. H_2_O_2_ activates PKA, which results in inhibitory phosphorylation of dynamin-related protein-1 (DRP1), a core component of the machinery involved in mitochondrial fission and linked with dynamic control of the cell cycle by mitochondria [54, 56]. Therefore, the localisation of DRP1 in relation to mitochondrial morphology was examined by confocal microscopy. Following treatment with N1, N2, DPI, and H89 (an inhibitor of PKA and, therefore, inhibitory phosphorylation of DRP1), mitochondrial morphology appeared more punctate (Fig. 4a). Whilst no significant increase in DRP1 pixels localising to whole mitochondria was observed (Fig. 4b), DRP1 clustering at the ends of the puncta was qualitatively more apparent than seen in control cells (Fig. 4a). Measurement of mitochondrial morphology showed a significantly reduced mitochondrial size, perimeter, and length with greater mitochondrial circularity in cells treated with N1 and N2 (Fig. 4c–f). A decrease in the percentage of the cytosol occupied by mitochondria was also seen for N2 (Fig. 4g) consistent with the decrease in TOM22 previously observed following treatment (Fig. 2j, k). The increase in punctate mitochondrial morphology was also observable in live cells using Mitotracker-Green staining (Sup Fig 3). After 2 h, a small but significant decrease in detectible DRP1 was also noted for N1 and N2 (Fig. 5a, c) and this was maintained at 24 h (Fig. 5b, c). Decreased DRP1 detection might indicate that this protein is undergoing increased turnover to maintain homeostasis. No change was observed for the mitochondrial fusion protein, mitofusin-1 (MFN1), at 24 h in response to N2 or DPI (Fig. 5d, e).

### N1 and N2 cause changes in SOD2 expression and SOD activity

**Fig. 6:**
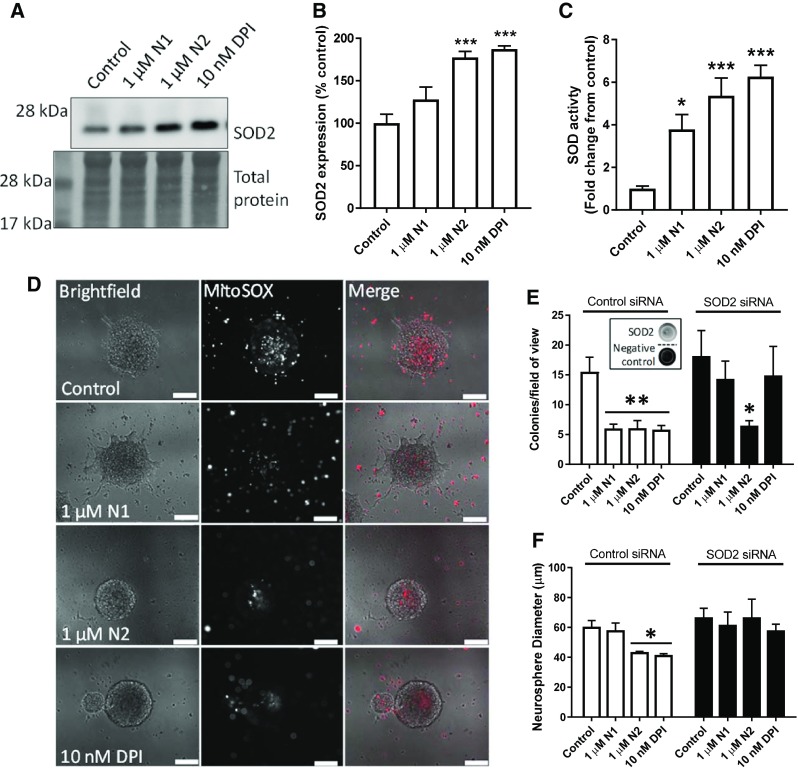
Decreased growth correlates with increased SOD2 protein and activity. **a** Immunoblots for SOD2 24 h following treatment with N1, N2 or DPI. **b** Densitometric quantification of **a**. *n* = 4. **c** SOD activity assay in cell lysates 24-h post-treatment with N1, N2 or DPI. *n* = 4. **d** Representative images of MitoSOX staining of whole-live neurospheres 24 h following treatment with N1, N2, or DPI. Scale bars = 50 μm. **e** Colony counts of the NCFA where NSCs were incubated with and without N1 and N2 and with siRNA targeting SOD2 expression or a control non-silencing siRNA (inset shows representative dots of SOD2 detection after knock-down). *n* = 3. **f** Colony diameter as measured for the NCFA described in **e**. *n* = 3. All data within the figure are presented as mean and SEM with significance indicated by *p < 0.05, **p < 0.01, ***p < 0.001 from respective control values

To ascertain whether the changes in mitochondrial structure caused by the N1 and N2 peptides could also affect their redox balance, mitochondrial antioxidant defence and ROS production were evaluated. Superoxide dismutase-2 (SOD2) is the central mitochondrial antioxidant, and therefore, cellular levels of SOD2 were evaluated by western blotting. A significant increase in expression was seen following treatment with the N2 peptide and inhibition of Nox signalling with DPI (Fig. 6a, b). This was also reflected in the cellular total SOD activity, which showed significant increases in response to N1 as well as N2 and DPI (Fig. 6c). Following 24 h exposure to N1, N2, or DPI, staining of live neurospheres with MitoSOX (a mitochondrially localised fluorescent superoxide probe) showed that the production of mitochondrial superoxide is reduced throughout the sphere and in individual cells surrounding the sphere (Fig. 6d). Using siRNA to knock down SOD2 abrogated the effect of N1 and DPI on reducing colony formation in the NCFA but did not prevent N2 reducing the number of colonies formed (Fig. 6e) suggesting effects of N2 beyond SOD2. However, the size of the colonies produced when incubated with N2 was no-longer reduced (Fig. 6f).

### NSCs show altered PrP N-terminal detection depending upon whether they are actively growing or quiescent

**Fig. 7:**
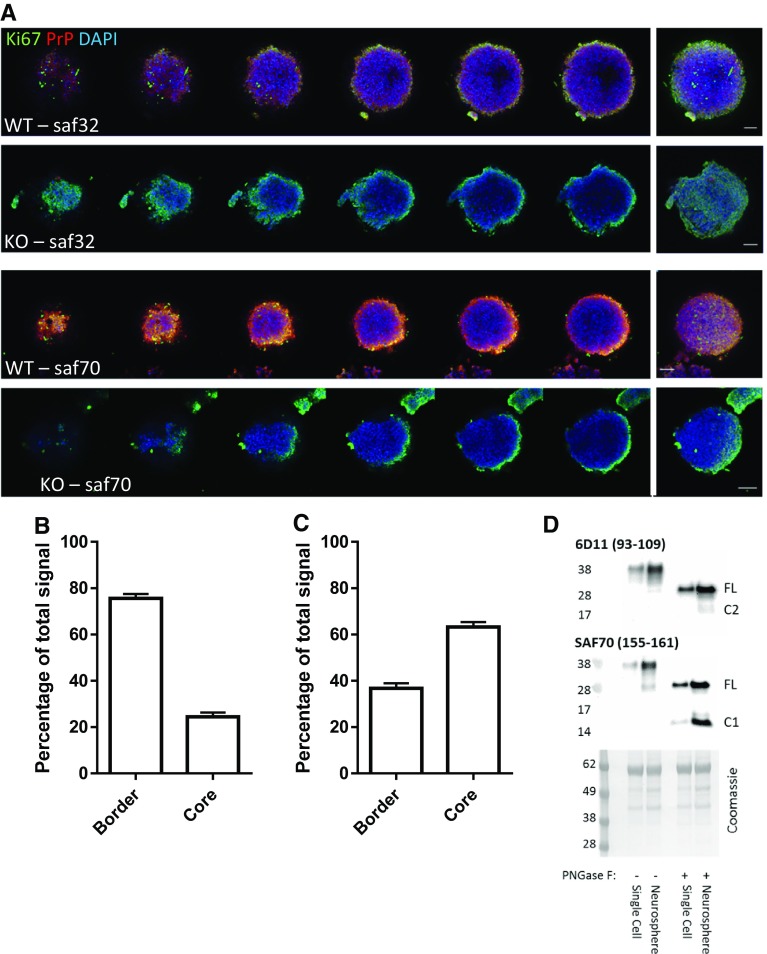
Characterisation of PrP cleavage in the quiescent core of neurospheres and in human brain precursor cells. **a** Representative confocal images of serial slices through a neurosphere and merger (right) when immuno-stained with Ki67 proliferation marker and N- (Saf32) or C- (Saf90) terminal PrP antibodies. Scale bar = 20 μm. **b** Quantification of peripheral and core staining of PrP as detected by Saf32. *n* = 3 independent repeats with a minimum of 20 spheres. **c** Quantification of peripheral and core staining of PrP as detected by Saf70. *n* = 3 independent repeats with a minimum of 20 spheres. **d** PNGase-F digests and PrP immunoblots comparing NSCs grown as neurospheres and those grown in single-cell suspensions

To ascertain whether endogenous cleavage of PrP correlates with NSC growth changes, we probed the profiles of N- and C-terminal PrP antibody reactivity in growing neurospheres (antibody-binding sites are shown in Fig. 1a). NSCs in the centre of a healthy neurosphere are quiescent, whereas those at the periphery are actively proliferating and this is reflected by the expression of the proliferation marker Ki67. The N-terminal fragments are rarely found in cell lysates but instead are detected as secretory fragments in conditioned media [31, 32] and, in addition, they have a predicted short cellular half life due to vulnerability to digestion. Therefore, while we cannot rule out saf32 (N-terminal) antibody reactivity due to the N-terminal fragments, the N-terminal signal in cells is more likely due to the presence of full-length, uncleaved PrP. The C-terminus of PrP has a predicted slow turnover post-cleavage and remains membrane anchored, permitting its detection in cells post-cleavage, and, therefore, saf70 (C-terminal) antibody reactivity could be due to full-length PrP, C1, or C2. N-terminal labelling of PrP was detected mostly at the periphery of the cultures, with only ~ 25% of the total signal detected in the core (Fig. 7a, b; individual channels and control nestin staining are shown in Sup Fig 4 and Sup Fig. 5, respectively). In contrast, significantly greater C-terminal labelling of PrP could be detected in the neurosphere core (Fig. 7a, c), indicating that more full-length PrP is found in cells that are actively growing and that PrP is predominantly cleaved in cells that are not growing. Cells at the periphery and within the core are shown at increased magnification in Sup Fig 6. Furthermore, when NSCs that were grown as neurospheres (where their core is quiescent) were compared with those grown as dissociated cells in suspension (where access to the mitogens in the media stimulates their growth) for 24 h, both the C1 and C2 cleavage fragments (detected after PNGase-F digest to remove N-linked complex glycans) were increased in the neurospheres (Fig. 7d).

## Discussion

**Fig. 8:**
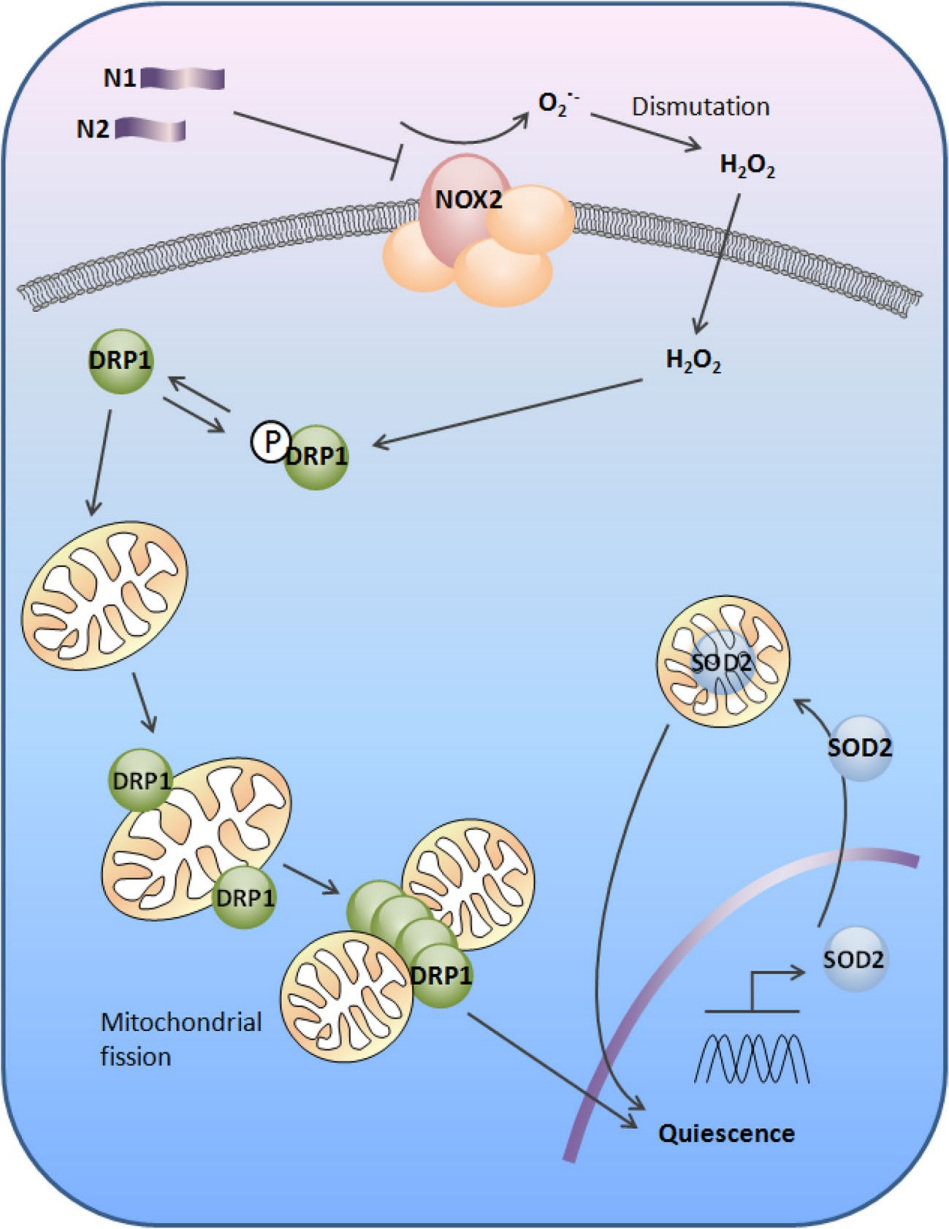
Schematic illustration of the redox signal transduction by N1 and N2 to induce quiescence in neural stem cells

Difficulty assigning a single or predominant function to PrP may have arisen, at least in part, from the numerous post-translational modifications to which it is subjected and the biochemically distinct protein/peptide species that are produced as a result. The data presented herein show that the N-terminal cleavage fragments, N1 and N2, can reduce intracellular redox signalling, with the N2 peptide directly reducing Nox catalytic activity. The reduced ROS increases the activity of DRP1, which mobilises to the mitochondria resulting in their fission within a few hours and later increasing protein levels of SOD2. The increased SOD2 levels and activity in turn maintain a low ROS environment within the mitochondria preserving cells in a quiescent state (Fig. 8).

The enzymes responsible for N1/C1 cleavage are still a matter of contention. It was first reported that A Disintegrin and metalloproteinase domain-containing protein 10 (ADAM10) was the candidate alpha-cleavage enzyme [31], although later work found that this enzyme was primarily responsible for secretory cleavage of PrP near the GPI anchor [58]. ADAM17 (alternatively known as tumour necrosis factor-α-converting enzyme [TACE]) and ADAM9 have also been implicated in N1/C1 cleavage, but a definitive identification has not yet been made; a detailed review of this work can be found in [59]. The N2/C2 cleavage event has been demonstrated to be mediated by ROS [60] and, during scrapie infection, by the calpain family [61]. Conditions that change the expression or activity of cleavage enzymes may shift the balance between full-length PrP and its cleavage fragments or shift the ratio of alpha- and beta-cleavage resulting in changed cellular outcomes. Therefore, the apparent cellular function of PrP may be as related to cellular protease expression levels as it is to total PrP levels.

The alpha-cleavage event producing N1 is generally considered constitutive, whereas the beta-cleavage event is thought to be primarily induced during disease [30] and caused by ROS. The N1 fragment, whilst demonstrating changes in each of the pathway components examined, was less potent in producing cellular changes than the N2 fragment and its potential to modulate Nox signalling less evident. The more aggressive action of N2 on this pathway may reflect differing functional requirements for the N1 and N2 fragments. For example, during times of stress, mitochondria fuse into tubular networks to avoid being destroyed by autophagy [55] and, in stem cells, autophagy becomes activated to prevent senescence [62]. Mitochondrial fusion is associated with G1-to-S-phase transition through the cell cycle [54], and therefore, the role of N2 in decreasing ROS signalling and maintaining a less fused mitochondrial population may function to preserve quiescence at times when the NSC pool is at risk of depletion. However, the converse could also be true that, as a result of N2 being more efficient at stimulating this pathway, such changes could be detrimental. Counteraction of mitochondrial fusion by N2 could lead to a loss of mitochondrial mass as mitochondria are engulfed by mitophagy, leaving cells struggling with energy demand. A slight decrease in mitochondria was seen for the N2 fragment, which might indicate that the fission event is causing a degree of mitochondrial loss even without the presence of damaging stress.

Alternative explanations may exist for the observed differences in biological effects between the N1 and N2 fragments. The N1 fragment contains a second charged cluster domain (resulting in charged domains at both ends of the peptide); this may endow it with extra properties such as the ability to bind and engage pathways that N2 cannot. Such putative-binding capacity may result in less N1 being available for modulation of the tested pathways. We have also observed aggregation of the 23-89P26/28A mutant in the previous studies [33] and the second charged domain within N1 may facilitate greater dimerisation/oligomerisation as compared with N2 thereby rendering less N1 peptide effectively available for interactions. Furthermore, only a single peptide concentration was compared in the current study; therefore, it is possible that higher concentrations of N1 could elicit the same intensity of reaction of the tested concentrations of N2. It is also unclear from the presented data why a difference should exist in cellular responses to N1 as a result of endogenous PrP expression; this could be related to the basal levels of N1 within these cells or lack of N1 and/or the pathways compensating for changed expression levels of the full-length protein. As the knock-out and Tga20 over-expressing NSCs demonstrate changed growth properties from the wild-type, which is especially apparent for the number of colonies generated by the Tga20 cells, basally changed pathways could influence how N1 is able to interact with and modulate cellular growth pathways.

PrP has been linked with increased ROS generated by Nox in the context of prion disease [52] and oxidative damage caused by overstimulation of Nox signalling was found to be mediated by interaction of the N-terminus at the cell membrane in an antibody-stimulated model of toxicity [51]. Tethering the N-terminus of PrP to the cell membrane reduces the capacity of PrP to undergo normal N2/C2 cleavage, as well as cellular resistance to oxidative stress [63], possibly by preventing the N2 fragment from modulating Nox function. How exactly the N-terminal fragments or N-terminus of full-length PrP interact with Nox is unknown. Both our previous in vitro studies that indicated no intrinsic antioxidant activity of N2 [33] and, herein, the decreased utilisation of NADPH substrate in response to N2 argue against an indirect ‘mopping up’ of the superoxide radicals. Other possibilities include direct interaction with one or more of the Nox subunits or modulation of the lipid membrane environment in which the Nox complex forms. The latter is a reasonable possibility as we have shown that both of these peptides bind to lipids under specific conditions [64, 65] and changes in the lipid environment are observed during N2 protection against oxidative stress [46].

From the data presented, we cannot rule out a direct effect of the peptides on the mitochondria themselves. Whilst N1 did not significantly change Nox activity or cellular SOD2 expression it did alter mitochondrial fission and knocking down SOD2 completely counteracted the effect of this peptide on cellular growth. Of interest, it has recently been reported that a population of PrP resides in the mitochondria of normal disease-free cells and is endoproteolytically cleaved similar to total brain PrP [66]. This might offer the N-terminal peptides, especially N1 as the predominant N-terminal cleavage product, unhindered access to the mitochondria from inside the cell, directly facilitating an influence of the peptide on this organelle. Consequently, this might also have a bearing on the apparent lower efficacy of N1 in stimulating these responses. We have previously shown that N2 traffics from the cell surface to the mitochondria [41], but this has not, to our knowledge, been examined for N1. The N1 fragment may be more functionally effective in stimulating quiescence if generated at the site of action (the mitochondria) rather than when it requires trafficking from the cell surface.

The role of SOD2 in regulating the cell cycle has become well characterised [67, 68]. Increased cellular levels of SOD2 are known to facilitate cellular transition to quiescence, whereas loss of SOD2 protein or increased superoxide signalling favours increased cellular growth [69, 70]. PrP has additionally been linked with SOD2 in the context of prion pathogenesis. During prion disease or prion infection of cells, changes of expression and activity of the SOD family of enzymes are seen, including a decrease in SOD2 protein levels [45, 71]. Correspondingly, neurogenesis during prion disease increases [39]. A further point to note when considering an influence of PrP-mediated signalling on SOD2 is that aberrations in SOD2 expression are linked with the uncontrolled cell cycling of cancer cells [68, 72]. Recently PrP expression has been linked with several cancers as well as the risk of metastases [73–75]. In addition to changes in mitochondrial SOD2, changes in mitochondrial fission and fusion have been linked with the uncontrolled cell cycling during cellular adaptation to the energy demands of cancer [76]. Regulation of SOD2 expression and mitochondrial dynamics by the N-terminal cleavage fragments of PrP or putatively the N-terminus in association with the full-length protein may explain why such disparate maladies are linked with this protein.

From our data, it would appear that detection of full-length PrP is predominantly observed in proliferating cells or cells residing alongside those that are actively proliferating, with quiescent cells staining only for C-terminal PrP. This might suggest that the signals stimulated by the N-terminal fragments can function in an autocrine or paracrine manner. Alpha-cleavage of PrP has been demonstrated to increase when PrP homodimerises at the cell membrane [77] and this may represent a mechanism for initiating intra/intercellular signalling. Questions remain as to the actions of full-length PrP, secretory (GPI-anchorless) PrP, C1, C2, or gamma cleaved PrP fragments in this pathway or cross-interactions with N1 and N2, but it seems highly likely that these proteins will be functional in their own right, bringing many more layers of complexity to the PrP function narrative.

Cross-talk between cellular redox signalling pathways and the mitochondria, and between the mitochondria and the nucleus, have been the subject of much study, but the role of the PrP N-terminal cleavage fragments as upstream modulators of these pathways has not previously been considered. As increased PrP expression levels are linked with increased growth of NSCs and herein we have linked the N-terminal cleavage fragments with reduced cell growth/increased cell quiescence, dynamic modulation of PrP cleavage may be part of an important cellular homeostatic mechanism. The modulation of redox signal transduction appears to be the first event influenced by the PrP cleavage fragments, after which the reduced intracellular ROS indicates that cells should enter a resting phase of life as modulated and maintained by their mitochondria. The diversity of the potential outcomes resulting from up-steam regulation of central redox signalling pathways in different cell types could have wide-reaching implications for many of the reported functions of PrP as this small protein begins to reveal its functional complexity.

## Electronic supplementary material

Below is the link to the electronic supplementary material.

Supplementary material 1 (PDF 124 kb)

Supplementary material 2 (PDF 197 kb)

Supplementary material 3 (PDF 326 kb)

Supplementary material 4 (PDF 83 kb)

Supplementary material 5 (PDF 95 kb)

Supplementary material 6 (PDF 77 kb)

Supplementary material 7 (DOCX 14 kb)
