# Supplementary material for: Prion protein cleavage fragments regulate adult neural stem cell quiescence through redox modulation of mitochondrial fission and SOD2 expression
Source: Cell Mol Life Sci. 2018 Mar 24;75(17):3231–49. doi: 10.1007/s00018-018-2790-3 (PMC6063333; doi:10.1007/s00018-018-2790-3)

Supplementary Figure 1. *RNAi control data.* Example western blots of cells silenced with siRNA targeting SOD2 (**A**) and Nox2 (**C**) as compared with non-silencing control siRNAs and quantifications of dot blots spotted from the cells used in the experiments within the main text (SOD2, **B**; Nox2, **D**). Data are shown as mean and SEM, and significance is indicated by *p<0.05.

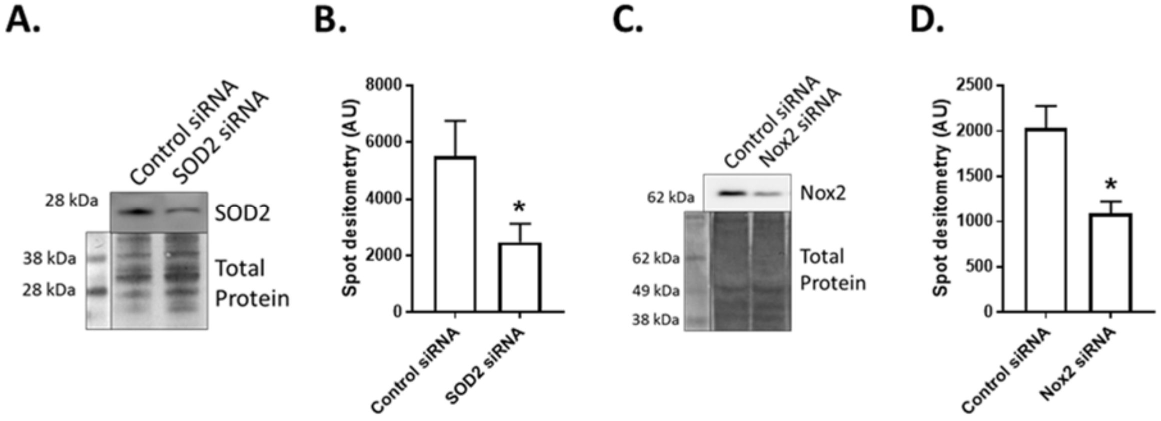

Supplement: Supplementary file 1 — Supplementary material 1 (PDF 124 kb) [file 18_2018_2790_MOESM1_ESM.pdf]
