# Supplementary material for: Prion protein cleavage fragments regulate adult neural stem cell quiescence through redox modulation of mitochondrial fission and SOD2 expression
Source: Cell Mol Life Sci. 2018 Mar 24;75(17):3231–49. doi: 10.1007/s00018-018-2790-3 (PMC6063333; doi:10.1007/s00018-018-2790-3)

Supplementary Figure 2. *Western blotting of signal protein phosphorylation.* Cells treated with 1 µM N2, or 10 nM DRP1 (Nox inhibitor) for 15 minutes were western blotted for phosphorylation of central signal transduction intermediates. **A.** Example images of blots. **B.** quantification of each phosphoprotein, total protein expression and the ratio of phospho:total protein shown as mean and SEM, n = 4.

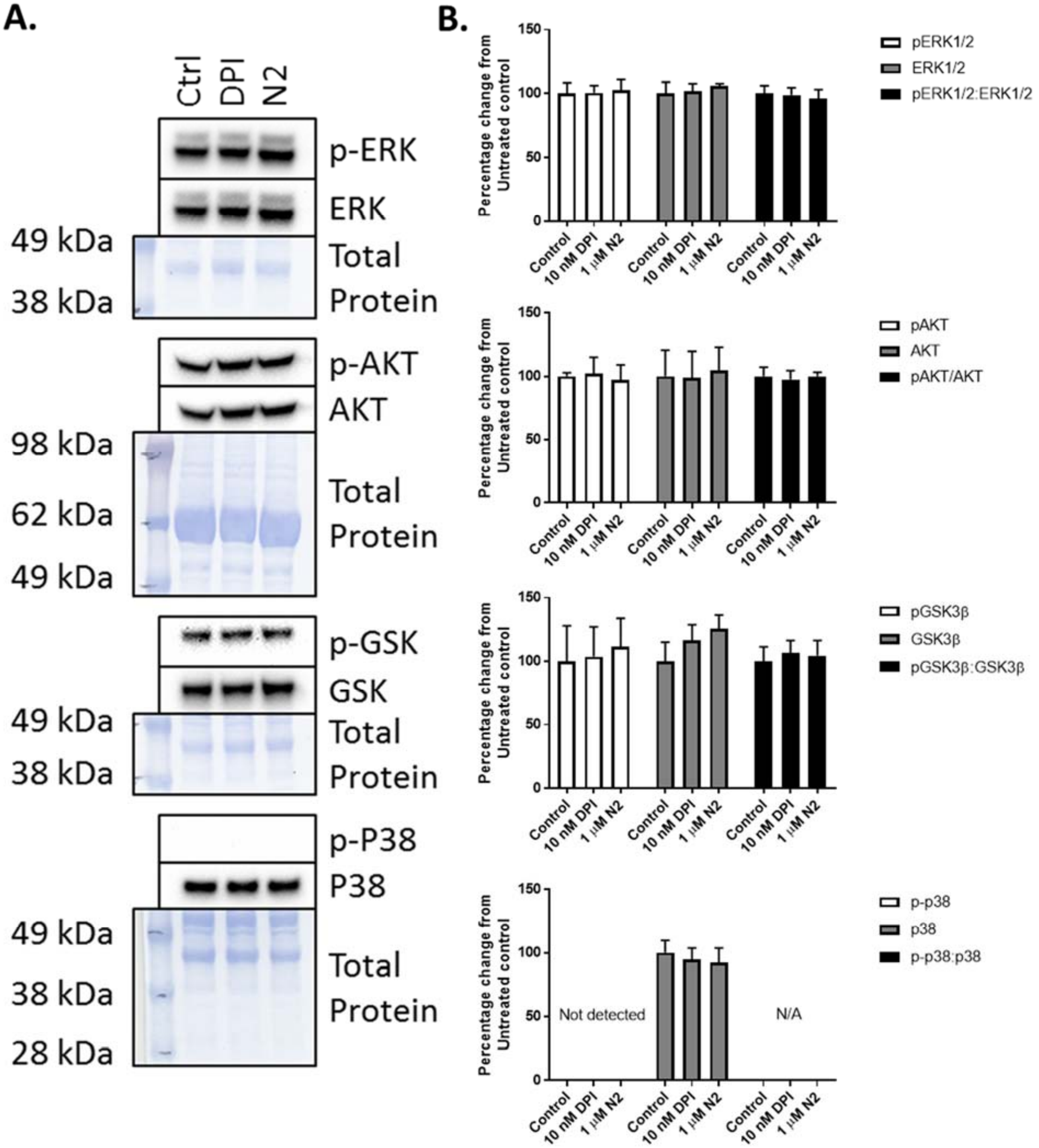

Supplement: Supplementary file 2 — Supplementary material 2 (PDF 197 kb) [file 18_2018_2790_MOESM2_ESM.pdf]
