# Supplementary material for: Prion protein cleavage fragments regulate adult neural stem cell quiescence through redox modulation of mitochondrial fission and SOD2 expression
Source: Cell Mol Life Sci. 2018 Mar 24;75(17):3231–49. doi: 10.1007/s00018-018-2790-3 (PMC6063333; doi:10.1007/s00018-018-2790-3)

Supplementary Figure 3. *Mitochondrial morphology following N1 and N2 treatment.* Cells were loaded with Mitotracker Green and treated with 1 µM N1, N2, or 10 nM DRP1 (Nox inhibitor) for 90 minutes before wide-field fluorescence imaging. Two representative images of each condition are shown. Scale bar = 15 µm.

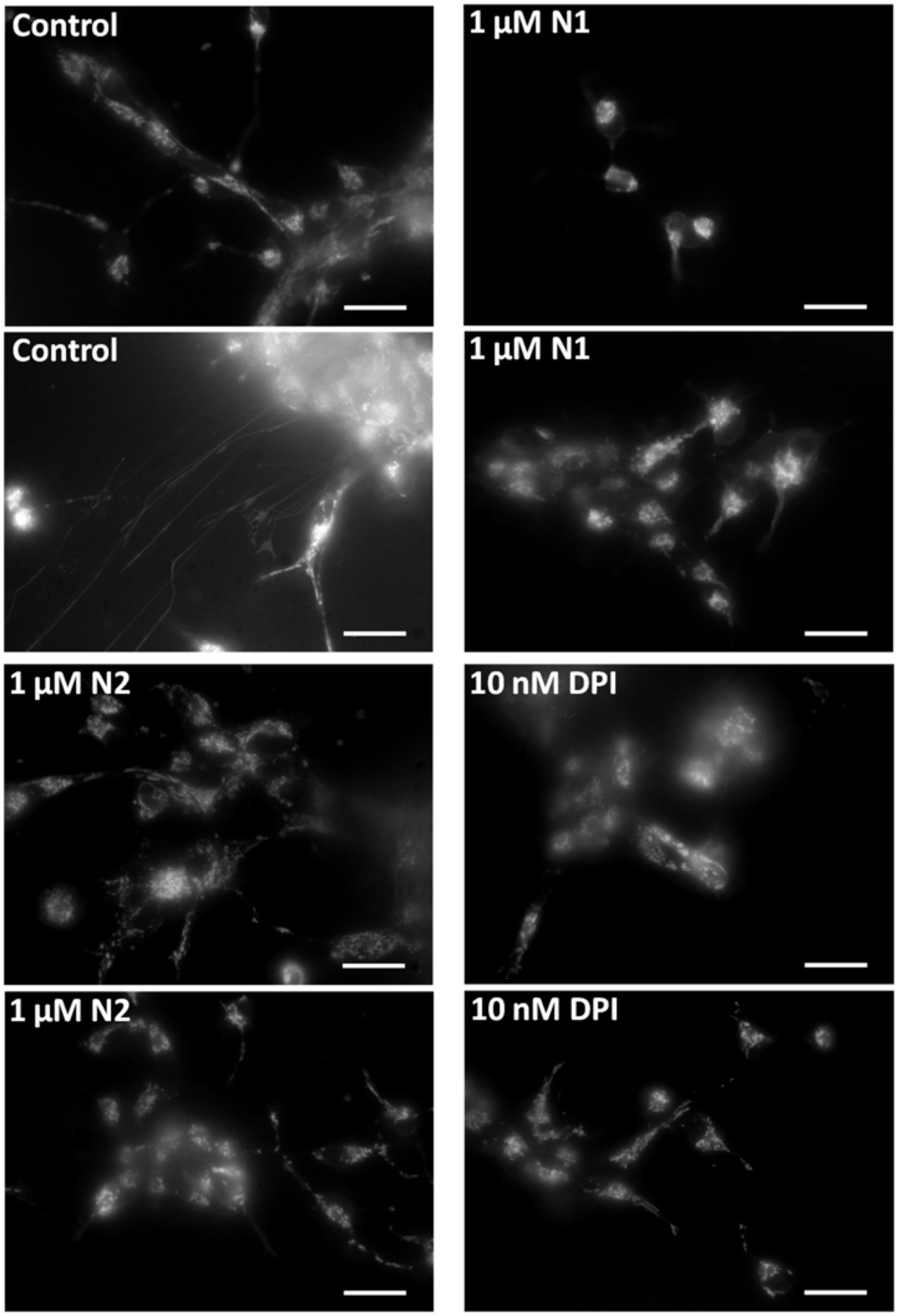

Supplement: Supplementary file 3 — Supplementary material 3 (PDF 326 kb) [file 18_2018_2790_MOESM3_ESM.pdf]
