# Supplementary material for: Prion protein cleavage fragments regulate adult neural stem cell quiescence through redox modulation of mitochondrial fission and SOD2 expression
Source: Cell Mol Life Sci. 2018 Mar 24;75(17):3231–49. doi: 10.1007/s00018-018-2790-3 (PMC6063333; doi:10.1007/s00018-018-2790-3)

Supplementary Figure 5. *Nestin control staining of 3D neurospheres.* Neurospheres were stained with nestin, as a marker that should uniformly label them. Confocal imaging shows that antibody penetration in the 3D staining reaches the core of the neurosphere.

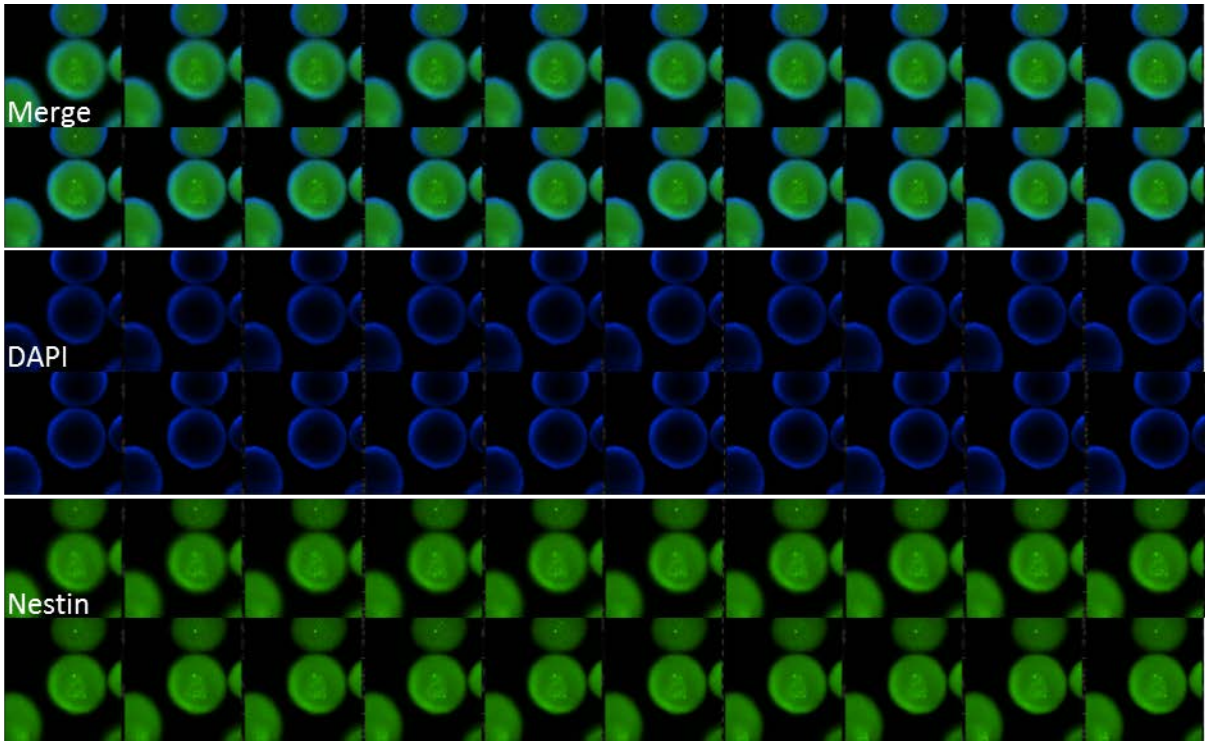

Supplement: Supplementary file 5 — Supplementary material 5 (PDF 95 kb) [file 18_2018_2790_MOESM5_ESM.pdf]
