# Supplementary figures and images for: Prion protein cleavage fragments regulate adult neural stem cell quiescence through redox modulation of mitochondrial fission and SOD2 expression
Source: Cell Mol Life Sci. 2018 Mar 24;75(17):3231–49. doi: 10.1007/s00018-018-2790-3 (PMC6063333; doi:10.1007/s00018-018-2790-3)

*Supplementary Figure 6. Higher magnification of neurosphere images shown in 6A.*

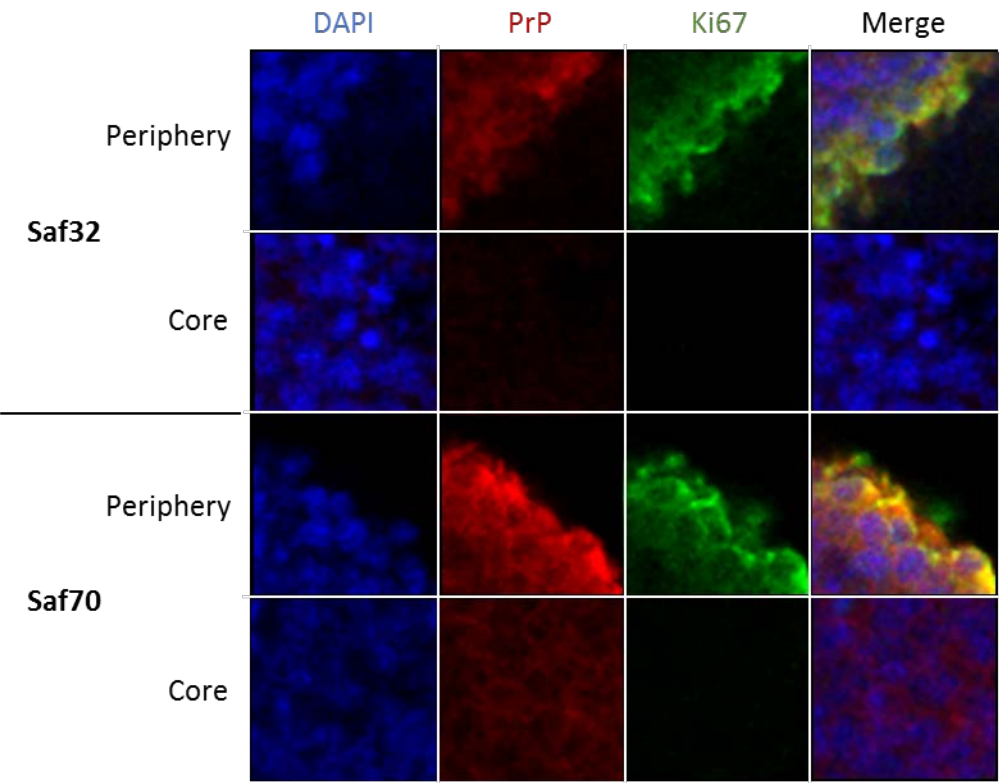

Supplement: Supplementary file 6 — Supplementary material 6 (PDF 77 kb) [file 18_2018_2790_MOESM6_ESM.pdf]
