# Supplementary material for: Prion protein cleavage fragments regulate adult neural stem cell quiescence through redox modulation of mitochondrial fission and SOD2 expression
Source: Cell Mol Life Sci. 2018 Mar 24;75(17):3231–49. doi: 10.1007/s00018-018-2790-3 (PMC6063333; doi:10.1007/s00018-018-2790-3)
Supplement: Supplementary file 7 — Supplementary material 7 (DOCX 14 kb) [file 18_2018_2790_MOESM7_ESM.docx]

*Supplementary Table 1. Antibody data.* The antibodies used within the study, their sources and targets and the concentrations used for western blotting and immunofluorescence staining (along with the secondary antibody concentrations) are listed.

| Antibody Info | Target Epitope | Western Blotting | | Immunofluorescence | |
| --- | --- | --- | --- | --- | --- |
|  |  | **Primary** | **Secondary** | **Primary** | **Secondary** |
| Pin1  Invitrogen (39-4700) |  | 1:1000 | 1:2000 |  |  |
| p21  Abcam (ab109199) |  | 1:1000 | 1:2000 |  |  |
| MFN1  Abcam (ab57602) |  | 1:1000 | 1:5000 |  |  |
| TOM22  Abcam (ab134274) |  | 1:1000 | 1:5000 | 1:50 | 1:250 |
| pERK1/2  Cell Signaling Technologies (4370) | Phospho T202/Y204 | 1:2000 | 1:5000 |  |  |
| ERK1/2  Cell Signaling Technologies (4695) |  | 1:1000 | 1:2000 |  |  |
| p-p38  Cell Signaling Technologies (4511) | Phospho T180/Y182 | 1:1000 | 1:2000 |  |  |
| p38  Cell Signaling Technologies (8690) |  | 1:1000 | 1:2000 |  |  |
| pAKT  Cell Signaling Technologies (4060) | Phospho S473 | 1:1000 | 1:2000 |  |  |
| AKT  Cell Signalling Technologies (4691) |  | 1:1000 | 1:2000 |  |  |
| Nox2  Abcam (ab129068) |  | 1:1000 | 1:2000 | 1:50 | 1:250 |
| Saf32  Sapphire Biosciences (189720) | Octameric repeat region (amino acids ~50-90) | 1:5000 | 1:10000 | 1:200 | 1:250 |
| Saf70  Sapphire Bioscience (189770) | Amino acids 142-160 | 1:5000 | 1:5000 | 1:200 | 1:250 |
| SOD2  Abcam (ab13533) |  | 1:5000 | 1:5000 |  |  |
| Nestin  Sigma-Aldrich (N5413) |  |  |  | 1:50 | 1:250 |
| Ki67  Abcam (ab15580) |  |  |  | 1:50 | 1:250 |
| Doublecortin (DCX)  Abcam (ab18723) |  |  |  | 1:100 | 1:250 |
| DRP1  Abcam (ab184247) | Amino acids 1-350 | 1:1000 | 1:2000 | 1:50 | 1:250 |
| pGSK  Cell Signaling Technologies (5558P) | Phospho S9 | 1:1000 | 1:2000 |  |  |
| GSK  Cell Signaling Technologies (9832S) |  | 1:1000 | 1:2000 |  |  |
